# Rehabilitation in Systemic Lupus Erythematosus With Class 4 Lupus Nephritis Secondary to Pneumonia: A Case Report

**DOI:** 10.7759/cureus.50889

**Published:** 2023-12-21

**Authors:** Trupti P Loya, H V Sharath, Neha Arya, Reva D Rajurkar, Nandini C Baheti

**Affiliations:** 1 Department of Paediatric Physiotherapy, Ravi Nair Physiotherapy College, Datta Meghe Institute of Higher Education and Research (DU), Wardha, IND

**Keywords:** rehabilitation, pneumonitis, pneumonia, lupus nephritis, systemic lupus erythematosus

## Abstract

We herein report an undisclosed case of systemic lupus erythematosus (SLE) with class 4 lupus nephritis (LN). It is an autoimmune disease that occurs when the body's immune system attacks its tissues. It results in significant tissue damage and inflammation in the afflicted organs. It may affect the kidneys, brain, lungs, skin, joints, and blood vessels. A 30-year-old female presented to Acharya Vinoba Bhave Rural Hospital (AVBRH) with the complaint of breathlessness, cough with expectoration, and fever for two months. The patient is having musculoskeletal renal difficulties and psychological effects. The objective is to reduce the symptoms and to improve the quality of life. A multidisciplinary treatment approach is used, which includes physiotherapy intervention and patient education. In conclusion, this case report mainly focuses on a multidisciplinary treatment approach to improve patient outcomes and quality of life.

## Introduction

Systemic lupus erythematosus (SLE) is the most common type of lupus. An autoimmune condition known as SLE develops when the body's immune system targets its own tissues. In the affected organs, it causes severe tissue damage and inflammation. Affected organs and tissues include the blood vessels, skin, joints, kidneys, brain, and lungs. Although there is no known cure for lupus, it can be controlled with medication and dietary changes [1]. In the United States, the incidence of lupus per 100,000 person-years was 5.1; it was higher in female than in males. With SLE estimates for males and females, the American Indian/Alaska Native population has the highest rate for men. SLE can affect people of any age, including youngsters. Fortunately, women who are capable of having children and are between the ages of 15 and 44 have the highest chance of developing SLE [2]. Four out of the 11 elements required by the European League Against Rheumatism (EULAR) and the American College of Rheumatology (ACR) collaborated to develop new classification criteria for SLE in 2019. These criteria are designed to improve the accuracy of SLE classification and aid in the identification of patients for inclusion in clinical trials. The new criteria that were published in Arthritis and Rheumatology in 2019 were needed to classify SLE. The following 11 criteria were present: photosensitivity, discoid rash or malar rash, oral or nasal sores, alopecia, non-erosive arthritis affecting two or more peripheral joints, serositis, renal disease, hematologic disease (hemolytic anemia), neurologic disease (seizures or epilepsy), immunologic criteria (antiphospholipid antibodies present based on either an abnormal serum level of IgM or IgG anticardiolipin antibodies or a tested positive result for lupus anticoagulant or anti-DNA antibody or anti-Sm antibody or false-positive syphilis test with Venereal Disease Research Laboratory (VDRL) or rapid plasma reagin (RPR) test), and antinuclear antibody positivity in the absence of drugs known to cause drug-induced lupus [3].

The treatment of people with pain has benefited greatly from physical therapy. It is up to the physical therapist to delve more into the many neurophysiological theories that have been put out to explain how physical and cognitive behavioural approaches to pain modulation work [4]. Exercise and routine medical treatment are contrasted with a placebo. Data from a single small trial comparing full-body vibration exercise to whole-body placebo vibration exercise suggest that exercise may have little to no effect on tiredness, functional ability, and discomfort [5]. In comparison to passive controls, patients with SLE were less physically fit, with decreased exercise capacity, decreased muscle strength, higher fatigue, and greater impairment. The overall management of fatigue and impairment in SLE should take into account interventions designed to cure depression and enhance aerobic fitness [6]. Dyspnea and cough are the main symptoms of hypersensitivity pneumonitis (HP), a lung condition brought on by inhaling an antigen to which the patient has already developed a reaction. The most active forms of HP are acute and subacute; nevertheless, the illness can persist and become chronic. HP may progress to end-stage lung disease [7]. Pneumonia continues to be the leading cause of death for both older people in wealthy countries and young children in developing ones. Numerous bacteria have been related to pneumonia, but a recent study has focused on the importance of viruses as pathogens. Concerns over the increasing incidence of viruses as causes of pediatric pneumonia have been raised by the vaccination programs' extensive use of the pneumococcal conjugate vaccine and the *Haemophilus influenzae* type B vaccine [8].

## Case presentation

Patient information

**Figure 1:**
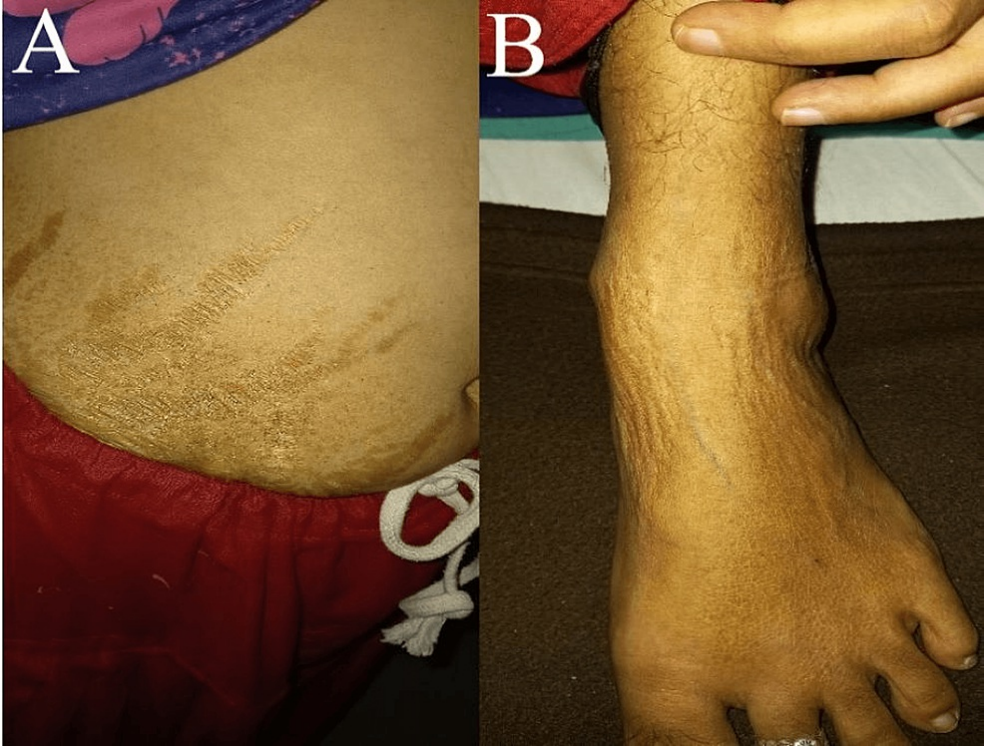
A: Rash seen on the lower abdomen. B: Rash seen on the anterior aspect of the left lower limb

A 30-year-old presented to Acharya Vinoba Bhave Rural Hospital (AVBRH) with a complaint of breathlessness, cough with expectoration, and fever for two months. Breathlessness was progressive, which aggravates on activity and relieves on rest and medication with grade 4 on Modified Medical Research Council (MMRC). The cough was insidious in onset and productive in nature, which aggravates on activity and relieves on rest and medication. The fever was sudden in onset and intermittent in nature, which was relieved by medication. The patient also complained of generalized weakness and joint pain, which aggravates on activity and relieves on rest and medication, which is present for two months. Computed tomography (CT) scans, bronchoscopy, and complete blood count (CBC) were performed, and the patient was referred for physiotherapy. The patient was a known case of SLE with class 4 LN for seven years. She has a rash on her abdomen and ankle as shown in Figure 1 and lived in a rural place and was illiterate.

Clinical examination

The patient was well oriented, cooperative, and conscious and was vitally stable. As per the MMRC scale, the score was grade 4. Observatory finding reveals a normal chest shape, and the breathing pattern was thoracoabdominal. No usage of accessory muscles was seen. On auscultation, bilateral air entry was reduced, and fine crackles were heard in the upper and middle lobes. On motor examination, the strength of both upper and lower limbs was reduced. The grades of manual muscle testing (MMT) according to Medical Research Council (MRC) are shown in Table 1.

**Table 1:** MMT MMT: manual muscle testing; 3+: full range of motion against gravity; 4+: full range of motion against gravity with minimal resistance; 5+: full range of motion against gravity with maximal resistance

	Right		Left	
Shoulder joint	Pre treatment	Post treatment	Pre treatment	Post treatment
Flexors	3+	5+	3+	5+
Extensors	3+	5+	3+	5+
Abductors	3+	5+	3+	5+
Adductors	3+	5+	3+	5+
Elbow joint				
Flexors	4+	5+	4+	5+
Extensors	4+	5+	4+	5+
Hip joint				
Flexors	3+	5+	3+	5+
Extensors	3+	5+	3+	5+
Abductors	3+	5+	3+	5+
Adductors	3+	5+	3+	5+
Knee joint				
Flexors	4+	5+	4+	5+
Extensors	4+	5+	4+	5+
Ankle joint				
Plantar flexors	3+	5+	3+	5+
Dorsiflexors	3+	5+	3+	5+

Clinical diagnosis

The patient was investigated for a routine checkup. An X-ray of the chest was taken, and the right side indicates a slight pleural effusion. Additionally, bronchoscopy was also out, revealing secretions in the right bronchus' middle and upper lobes. On CT thorax examination, numerous soft tissue density nodules were observed in a random distribution, with some displaying a tree-in-bud appearance predominantly affecting the right middle and lower lobes. A few cavitary lesions are pointed out in the right lower lobe, the largest measuring 18x10 mm. Atelectatic bands are indicated in bilateral lower lobes and right middle lobe. The rest of the lung parenchyma appears normal after these investigations. The patient has been diagnosed with anemia, pneumonia, and pneumonitis for which she was on pharmacological treatment such as Shelcal 500 mg (Torrent Pharmaceuticals, Ahmedabad, India), Duolin inhaler (Cipla, Mumbai, India), and methylprednisolone.

Physiotherapy intervention

There was a split physiotherapy treatment to emphasize the significance of taking the advised course of action. First, the patient was informed about her condition and gave her informed consent for the course of therapy, as shown in Table 2.

**Table 2:** Physiotherapy treatment protocol ROM: range of motion; ACBT: active cycle of breathing technique; VO2 max: maximum rate of oxygen consumption; MPH: miles per hour

Sl. no.	Intervention objectives	Clinical intervention	Treatment protocol
1	Education of patient	Describe the treatment plan	At the outset of treatment, describe the treatment plan
2	To improve aerobic capacity	Six-minute walk test	Ambulation for six minutes
3	To reduce breathlessness	Pursed lip breathing, dyspnea-relieving position	10 reps x 3 sets
4	To improve the strength of respiratory muscle	Spirometry	10 reps x 2 sets three times/day
5	Airway clearance	ACBT flutter	5 cycles x 1 sets three times/day
6	To increase lung volume and functional residual capacity	Thoracic expansion exercise	10 reps x 2 sets three times/day
7	To maintain ROM and joint integrity	Exercises for the upper and lower extremities' bilateral active ROM	10 reps x 1 sets three times/day progressing to 10 reps x 2 sets four times/day
8	Endurance training	Treadmill walking and cycling	Treadmill: 10 minutes (speed increased, every 3 minutes by 1.7 mph). Cycling: 70% VO2 max

**Figure 2:**
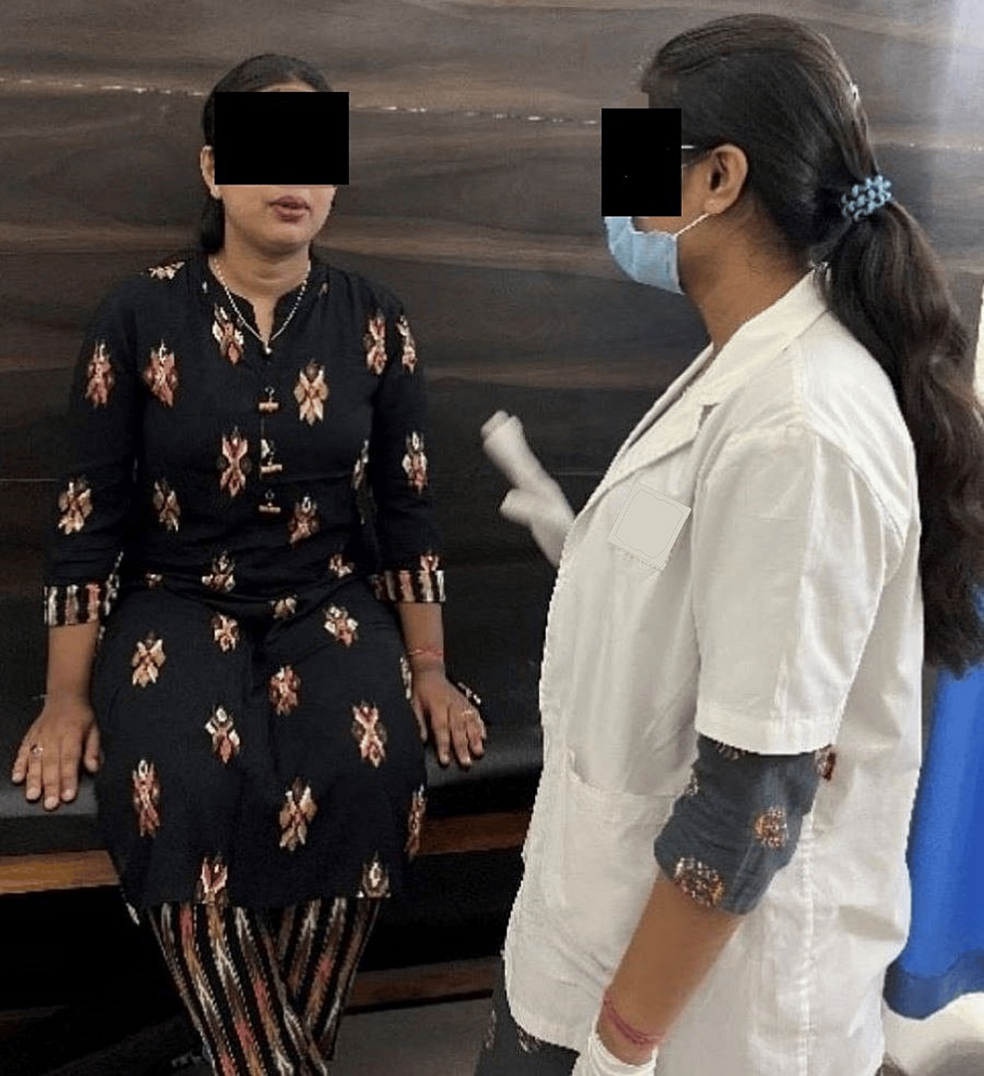
Patient performing pursed lip breathing

**Figure 3:**
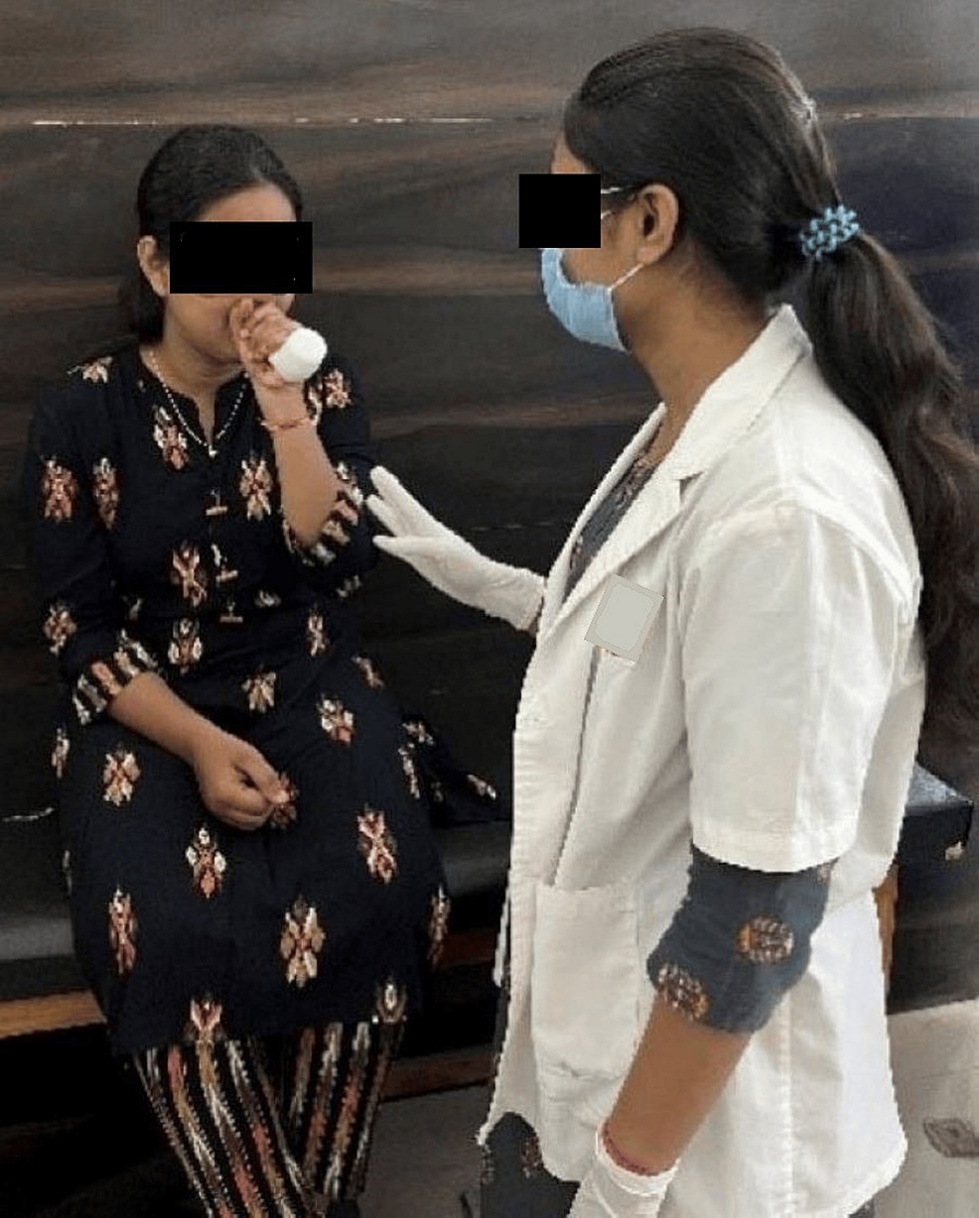
Patient using flutter

**Figure 4:**
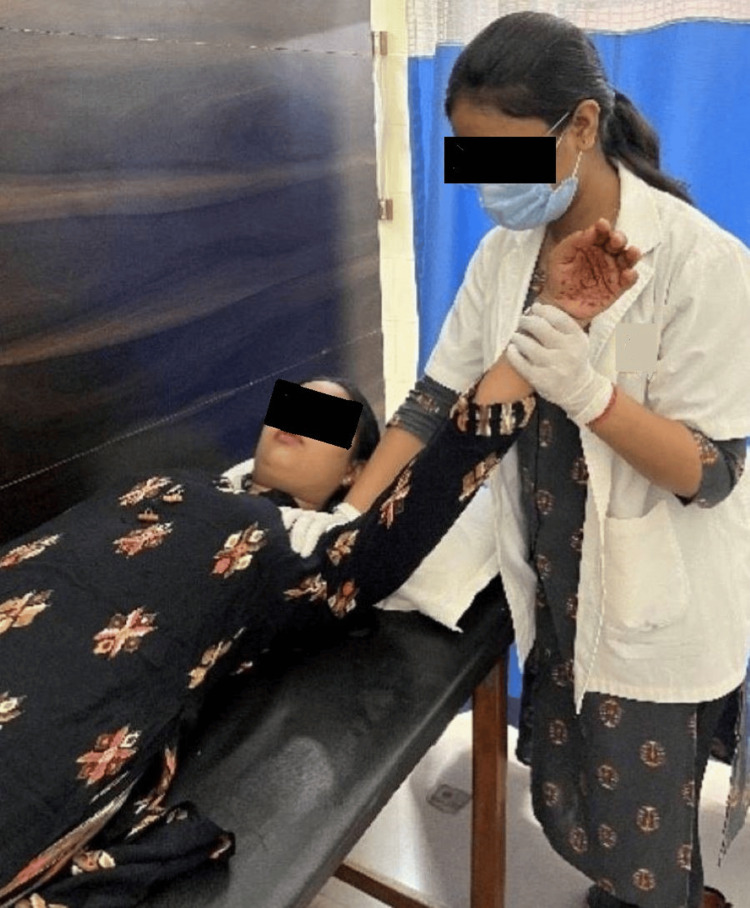
Therapist performing ROM exercises for an upper limb on the patient ROM: range of motion

Figure 2 depicts the patient performing pursed lip breathing. Figure 3 depicts the patient using flutter. Figure 4 depicts the therapist performing range of motion (ROM) exercises for an upper limb on the patient.

Outcome measures

For four weeks, exercises in thoracic expansion, spirometry, active cycle of breathing technique (ACBT), MMRC, and the six-minute walk test were conducted from which the patient's recovery was seen. Spirometry, chest expansion exercises, and pursed lip breathing all helped to lessen dyspnea. The six-minute walk test improved endurance. ACBT helped to eliminate secretions, as shown in Table 3.

**Table 3:** Outcome measures MMRC: Modified Medical Research Council; TLC: total lung capacity; MMRC grade 3: stops for breath after walking about 100 yards (91 meters) or after a few minutes on level ground; MMRC grade 1: gets short of breath when hurrying on level ground or walking up a slight hill; CC: cubic centimeter

Sl. no.	Outcome measures	Pre treatment	Post treatment
1	Six-minute walk test	Rate of perceived exertion: 7 distance, 290	Rate of perceived exertion: 3 distance, 420
2	MMRC dyspnea grading	Grade 3	Grade 1
3	Incentive spirometry	600 cc with three-second hold TLC: 1.8 liters	1200 cc with two-second hold TLC: 2.4 liters

## Discussion

In this study, physiotherapy interventions were applied to an SLE patient with cardiovascular and joint involvement. At the time of referral, the patient showed breathlessness, cough with expectoration, generalized weakness, and joint pain in all four limbs. After four weeks of treatment, the patient can now breathe efficiently, and the MRC grading has been modified to grade 5. Thus, the current results indicate that the physiotherapy interventions applied to the patient effectively recovered her cardiovascular and muscular function. Exercise for the upper and lower limbs considerably enhances hand function, pain, performance in everyday activities, and quality of life in people with SLE [9]. Programs for resistance and aerobic exercise exhibited definite advantages and were well tolerated by SLE patients with stable conditions [10]. Our first clinical goal was to make the patient breathe properly and decrease joint pain by giving aerobic exercises and manual muscle therapy. Being physically inactive is a major risk factor for cardiovascular events and is quite common in people with sickle cell disease [11,12].

In the general population, cardiovascular morbidity and mortality are reduced by physical activity and exercise [13,14]. High levels of physical activity (in individuals over 50) are linked to an enhanced life expectancy of 3.7 years for men and 3.5 years for women, according to data from the Framingham Heart Study [15]. It has been shown that physical activity significantly affects how well the immune system functions. However, frequent moderate-intensity exercise has been shown to have favourable benefits on the immune system, while extended periods of intense exercise training are thought to impair immune system function [16]. Regular, moderate-intensity exercise has been demonstrated to be "immuno-enhancing" and has been useful in improving immunocompromised patients' reactions to vaccinations. Regular moderate-intensity exercise may enhance the functioning of the immune system by reducing the levels of inflammatory factors, maintaining thymic mass, changing the makeup of younger and older immune cells, improving immune surveillance, and reducing psychological stress [17]. Above all, exercise training has become a viable therapy approach to address physical impairment in adults with SLE [18]. The following conditions have been found to be risk factors for severe forms: advanced age, chronic alcoholism, diabetes mellitus, heart disease, and a deficit in glucose-6-phosphate dehydrogenase [19,20].

## Conclusions

This case study emphasizes how important complete rehabilitation is to the management of class 4 LN in SLE. A multidisciplinary strategy that included individualized physical therapy and psychological support led to notable improvements in the patient's functional ability, pain control, and general quality of life. In addition to reducing the crippling symptoms of SLE, the effective integration of rehabilitation techniques also brought attention to the intricate interactions between the disease's psychological and medical components. To improve long-term outcomes and quality of life for people with SLE and LN, more research is necessary to investigate alternative rehabilitative modalities and create more focused interventions.
